# Combining Rosetta Sequence Design with Protein Language Model Predictions Using Evolutionary Scale Modeling (ESM) as Restraint

**DOI:** 10.1021/acssynbio.3c00753

**Published:** 2024-04-03

**Authors:** Moritz Ertelt, Jens Meiler, Clara T. Schoeder

**Affiliations:** † Institute for Drug Discovery, University Leipzig Medicine Faculty, Liebigstr. 19, D-04103 Leipzig, Germany; ‡ Center for Scalable Data Analytics and Artificial Intelligence ScaDS.AI, D-04105 Leipzig, Germany; § Department of Chemistry, Vanderbilt University, Nashville, Tennessee 37235, United States; ∥ Center for Structural Biology, Vanderbilt University, Nashville, Tennessee 37235, United States

**Keywords:** computational protein design, protein language models, Rosetta, evolutionary fitness, thermodynamic stability, *de novo* proteins, protein language model

## Abstract

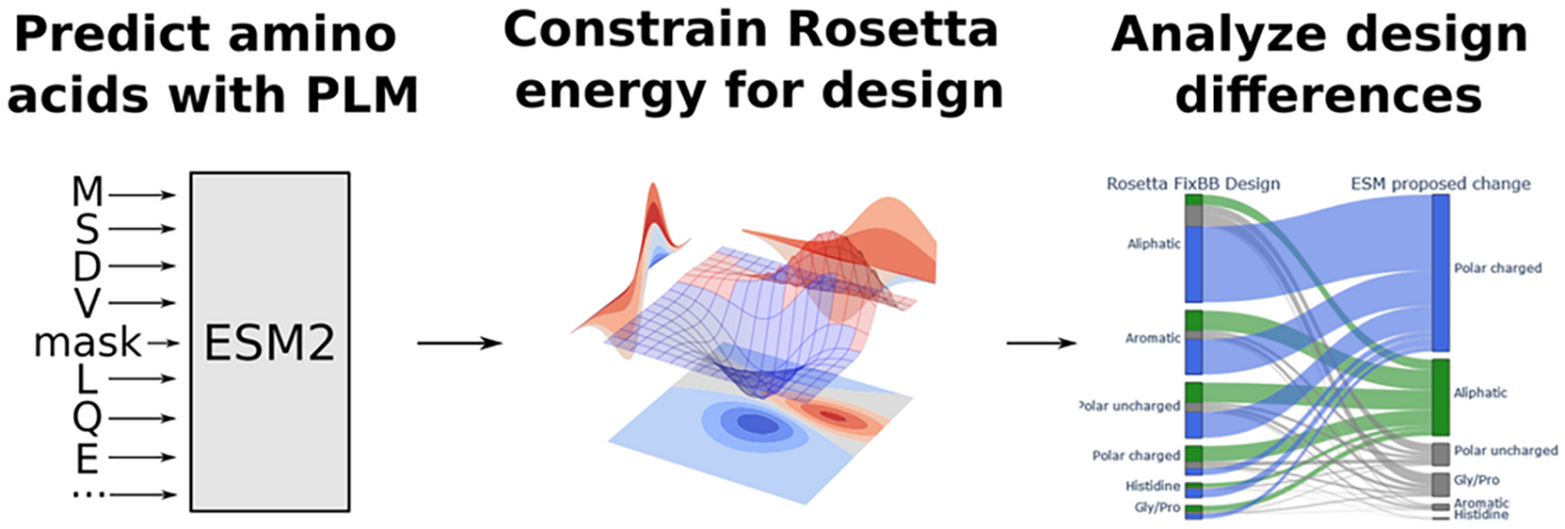

Computational protein sequence design has the ambitious goal of modifying existing or creating new proteins; however, designing stable and functional proteins is challenging without predictability of protein dynamics and allostery. Informing protein design methods with evolutionary information limits the mutational space to more native-like sequences and results in increased stability while maintaining functions. Recently, language models, trained on millions of protein sequences, have shown impressive performance in predicting the effects of mutations. Assessing Rosetta-designed sequences with a language model showed scores that were worse than those of their original sequence. To inform Rosetta design protocols with language model predictions, we added a new metric to restrain the energy function during design using the Evolutionary Scale Modeling (ESM) model. The resulting sequences have better language model scores and similar sequence recovery, with only a minor decrease in the fitness as assessed by Rosetta energy. In conclusion, our work combines the strength of recent machine learning approaches with the Rosetta protein design toolbox.

## Introduction

Computational protein design aims to create stable and functional proteins that can be utilized in a wide range of applications spanning from enzymes to biological therapeutics. Up until recently, protocols mainly based on biophysical principles were collected in the Rosetta software suite for molecular modeling and design.^1^ Leveraging the collective work of over 90 laboratories around the world has led to many successes including improved antibodies,^2^ novel enzymes for biotechnology^3^ and the first *de novo* protein.^4^ Nowadays, through the advance of hardware capabilities and seminal work in machine learning (ML), many of the “classic” modeling and design tasks are outperformed by large neural networks. The most phenomenal example is AlphaFold2,^5^ enabled through the tedious work of scientist depositing proteins structures to the Protein Data Bank (PDB),^6,7^ the critical assessment of protein structure prediction (CASP) competition,^8^ and progress in the use of machine learning technology, unlocking protein structure prediction for a large part of the protein universe.

Similarly, the success rates of designed protein sequences folding to their target structure have increased manifold through machine learning protocols like ProteinMPNN.^9^ Additionally, protein language models (PLMs) trained on millions of protein sequences in an unsupervised fashion have shown impressive performance on different downstream tasks, including protein contact and structure prediction.^10−13^ Their embeddings are a key component in recent state-of-the-art models for small molecule or protein–protein docking.^14,15^ The strength of PLMs is their embedding of evolutionary information in a high dimensional space, as seen by the ability to predict the evolutionary dynamics of diverse proteins.^16^ This ability has been leveraged for the evolution of antibodies,^17^ increasing stability and/or binding affinity, starting from a round of predicted single point mutations and their combinations. Nevertheless, there is existing complexity beyond the standard 20 letter amino acid code currently not captured by these ML models, ranging from post-translational modifications to noncanonical amino acids, for which there are available protocols in Rosetta.

Historically, informing protein design methods with evolutionary information was meant to limit the mutational space to more native (“natural”) sequences without highly deleterious mutations, as these mutations have been diminished by natural selection. The PROSS protocol,^18,19^ for example, combines the information from a multiple sequence alignment of the target protein represented as position-specific-substitution-matrix (PSSM)^20^ with Rosetta combinatorial sequence design to find optimal combinations of highly stabilizing mutations. Recently, Hie et al.^17^ showed that restricting the mutational space based on predicted PLM likelihoods has a similar effect of enriching for stable and functional proteins, without requiring the curation of evolutionary data.

In this work, we therefore set out to combine the strength of PLMs using the Evolutionary Scale Modeling (ESM) model family with the flexibility of Rosetta, enabling efficient combinatorial sampling of the PLM-predicted sequence space of proteins. To do so, we analyzed the predicted probability (PLM score) of Rosetta designed sequences of 34 *de novo* proteins, as those were not part of the PLM training data (all of them produce no hits against the UniProt database in a blast search). We found that Rosetta-designed sequences have PLM scores that are worse than those of the originally described (wild type) sequences of the *de novo* proteins. We analyze the predicted pitfalls of Rosetta fixed backbone design (FixBB) and LayerDesign, revealing the complexity of protein surface amino acid composition. To design protein sequences with native-like PLM scores, we added a PLM metric to Rosetta to score a given protein and create a position-specific-probability matrix used to restrain the Rosetta energy function during design. We show that the resulting sequences have better PLM scores and similar sequence recovery with little impact on the Rosetta total energy.

## Results and Discussion

### Rosetta Designed Sequences Have Worse Protein Language Model Scores than Their Native Sequences

**Figure 1:**
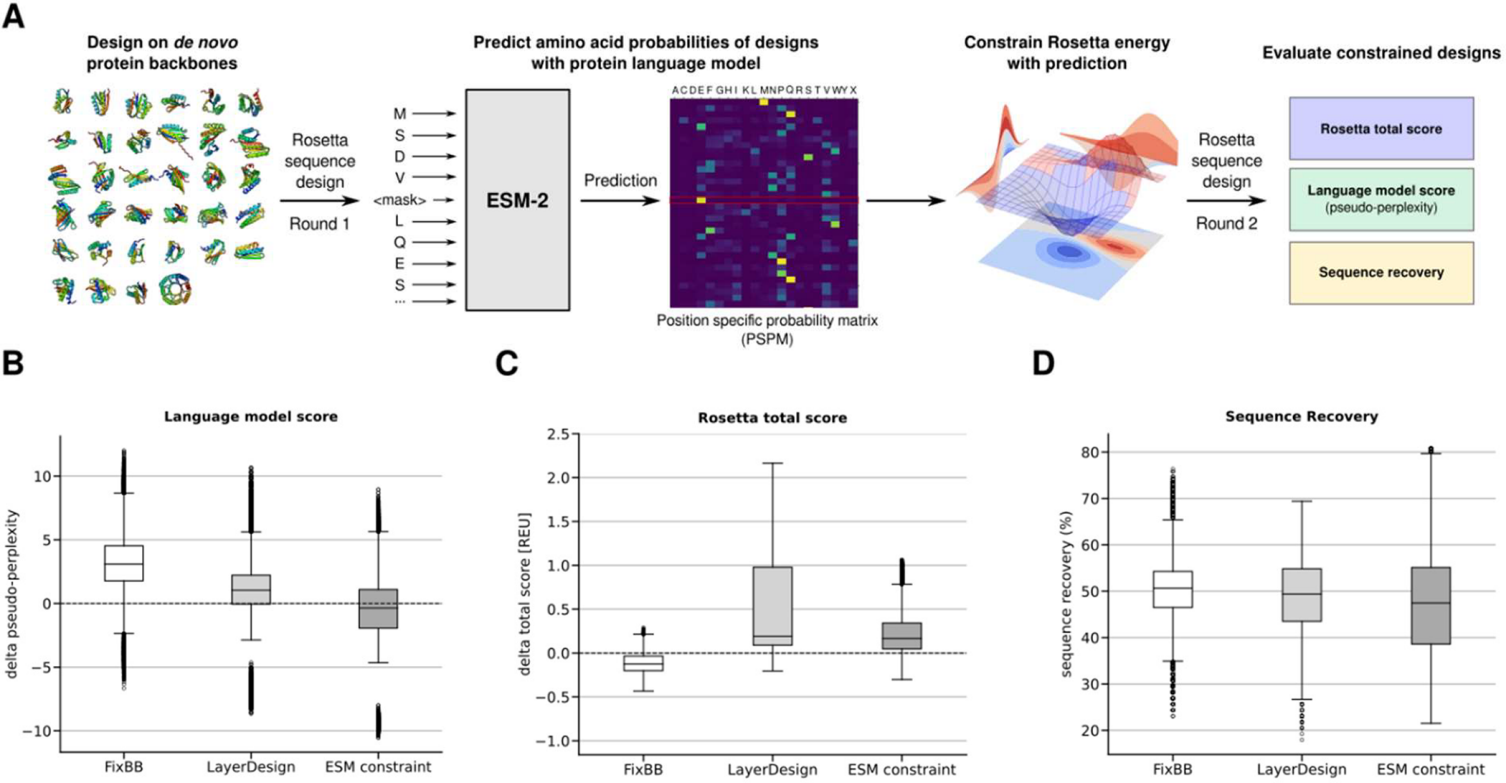
Protein sequence design workflow and resulting metrics. (A) Overview of the workflow for restraining the Rosetta energy function with ESM language model predictions. After an initial round of sequence design, the top scoring sequence is used to predict amino acid probabilities by using the ESM language model. The resulting probability matrix is used to restrain the Rosetta energy function before a second round of sequence design. (B) Language model score (pseudoperplexity) for the different design approaches normalized by the score of the respective native sequence. Here and in the following boxplots the median is indicated as a black line; boxes depict the interquartile range (IQR), whiskers represent 1.5 × IQR. (C) Rosetta total score of designs normalized by the respective native protein. (D) Sequence recovery of the different design approaches.

We first set out to test whether sequences designed by Rosetta have worse protein language model scores than their native counterparts. To do so, we selected a benchmark of 34 *de novo* proteins, as these proteins were not part of the original PLM training data and used Rosetta FixBB (one round of PackRotamersMover) to design 1000 new sequences for each protein. Subsequently, we scored the sequences using the pseudoperplexity of the ESM 2 language model,^11^ where sequences with lower values are seen as more probable and found that the Rosetta-designed sequences scored worse than their original sequences (Figure 1). The pseudoperplexity is the exponentiation of the average negative logarithm of the probabilities predicted by the PLM. Additionally, a correlation between the predicted language model likelihood of sequences and Rosetta total energy was absent (Table 1). Similarly, a recent study by Johnson et al. has shown that there is no clear correlation between ESM and Rosetta scores for a large set of enzyme mutations.^21^

**Table 1:** Correlation between Language Model Score (Pseudoperplexity) and Rosetta Energy Function Terms of Rosetta FixBB Design Sequences

score type	median Pearson *r*	st. deviation
fa_atr	–0.172235	0.146290
fa_dun_rot	–0.142004	0.166209
fa_sol	–0.134754	0.222276
fa_intra_sol_xover4	–0.119955	0.182549
lk_ball	–0.090473	0.191947
fa_intra_atr_xover4	–0.068135	0.158594
hbond_bb_sc	–0.056970	0.142858
fa_dun_semi	–0.041084	0.139465
p_aa_pp	–0.011742	0.183959
omega	0.013219	0.183884
fa_dun_dev	0.018607	0.122845
lk_ball_iso	0.021725	0.188231
fa_intra_rep_xover4	0.028418	0.149158
total_score	0.036711	0.173967
fa_rep	0.053064	0.165754
fa_intra_elec	0.056037	0.159373
lk_ball_bridge_uncpl	0.103547	0.143101
lk_ball_bridge	0.103822	0.141972
hxl_tors	0.107457	0.179690
hbond_sc	0.110155	0.173900
fa_elec	0.139519	0.168419
rama_prepro	0.231027	0.168586
ref	0.257970	0.222417

### Aliphatic Surface Residues Receive the Worst Protein Language Model Scores

**Figure 2:**
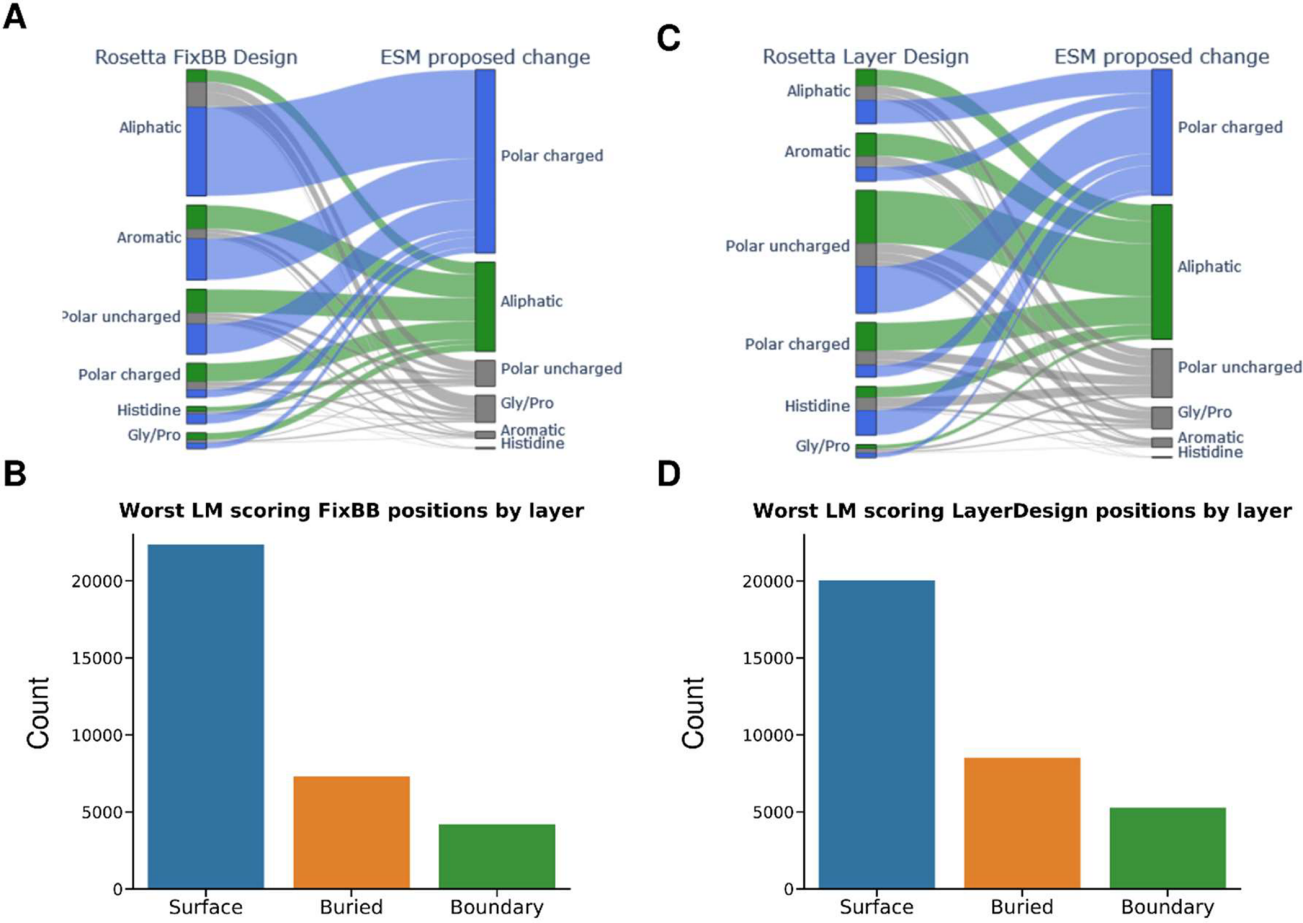
Worst protein language model score positions of Rosetta designs and their proposed replacement. (A) ESM language model (PLM) proposed changes to the ten worst positions by PLM score in Rosetta FixBB designs (34 000 positions in total). (B) FixBB worst scoring positions by layer classified into surface, boundary, and core depending on the solvent-accessible-surface-area (SASA). (C) ESM language model proposed changes to the ten worst scoring positions in Rosetta LayerDesign designs (34 000 positions in total). (D) LayerDesign worst scoring positions by layer classified into surface, boundary, and core depending on the solvent-accessible-surface-area.

Next, we set out to find the reason for the worse PLM scores of Rosetta sequence designs compared with their native counterparts. Therefore, we collected the ten positions with the worst PLM score of each designed sequence of each of the 34 benchmark proteins, as well as their predicted replacement (Figure 2A). Of these 34 000 positions, 9 065 were aliphatic residues (Ala, Ile, Leu, Met, Val) where the highest likelihood replacement was a polar charged residue (Arg, Lys, Asp, Glu). The next largest group was 4 206 aromatic amino acids (Phe, Tyr, Trp) again with polar charged residues deemed by the PLM to be the most probable instead. In general, for 17 917 of the 34 000 worst scoring positions polar charged residues were predicted to be most likely (not counting polar charged to polar charged proposed mutations). Additionally, over two-thirds of the worst scoring positions were located on the surface (Figure 2B).

### Restricting Amino Acid Mutations by Local Context Improves PLM Score

A common strategy to avoid composition biases in Rosetta is restricting the available amino acids at each position depending on local context with LayerDesign^22^ or reweighting the Rosetta energy function.^23,24^ The LayerDesign protocol classifies residues based on their solvent-accessible-surface-area (SASA) into core, boundary, or surface position and additionally considers their secondary structure. The LayerDesign rules are meant to prevent too many hydrophobic residues on the surface as well as too many polar residues in the core of a protein. We used LayerDesign to obtain 1000 sequences for each of the 34 *de novo* proteins and scored them using the ESM language model, showing a clear improvement in score over the FixBB designs but still worse than their native sequences (Figure 1B). As expected, restricting the available amino acids led to worse Rosetta energies compared to the FixBB protocol, while having similar sequence recovery (Figure 1C, D). We again analyzed the ten worst PLM score positions of the resulting LayerDesign outputs as well as their predicted replacement (Figure 2C). Interestingly, 5 238 of the 34 000 positions were polar uncharged residues (Gln, Asn, Thr, Ser, Cys) with a predicted aliphatic (Ala, Ile, Leu, Met, Val) replacement. The second largest fraction of positions were 4662 polar uncharged residues with polar charged residues (Arg, Lys, Asp, and Glu) predicted as more likely by the PLM. Again, almost two-thirds of the positions were located on the surface (Figure 2D).

### Restraining the Rosetta Energy Function with Protein Language Model Probabilities Results in Native-Like PLM Scores

Next, we restrained the Rosetta energy function with ESM predictions from the initial round of FixBB design. To do so, we used the FixBB designed sequence with the highest Rosetta total score for each protein as input for ESM and predicted the probability of each amino acid at each position, generating a position-specific-probability-matrix (PSPM). This PSPM serves as input for the FavorSequenceProfile mover which is commonly used to restrain the Rosetta energy function with evolutionary information based on a multiple sequence alignment.^18^ Subsequently, we performed another round of sequence design (PackRotamersMover) with the restrained energy function, again generating 1 000 sequences for each protein. As expected, the resulting sequences have PLM scores comparable to those of their native sequences (Figure 1C). Furthermore, the impact on the unrestrained Rosetta total score is lower than the LayerDesign protocol while showing similar sequence recovery (Figure 1D).

**Figure 3:**
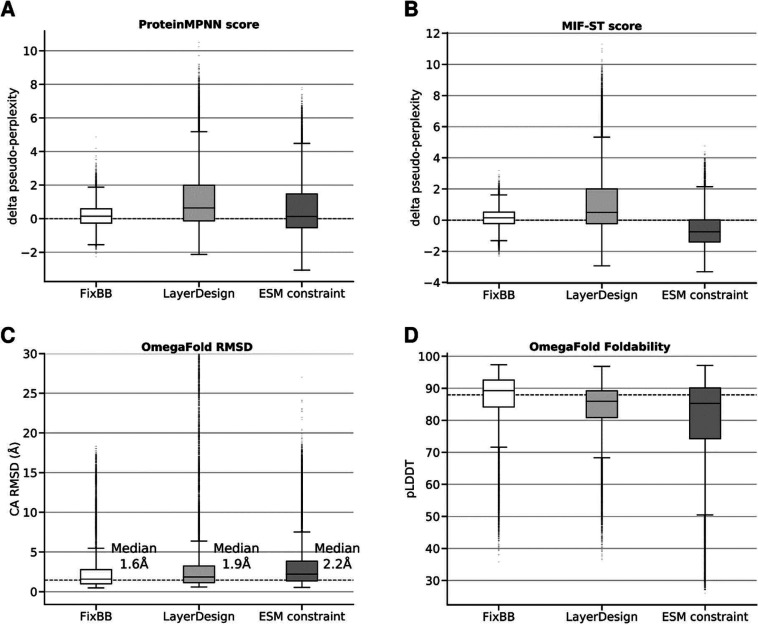
*In silico* validation of all designed sequences (34 000 for each approach). (A) ProteinMPNN score (pseudoperplexity) for the different design approaches normalized by the score of the respective native sequence. (B) Masked inverse folding with sequence transfer (MIF-ST) score (pseudoperplexity) for the different design approaches normalized by the score of the respective native sequence. (C) Root-mean-square deviation (RMSD) in Ångström (Å) between C-alpha atoms of designed structures (OmegaFold prediction) and target structure. The dashed line presents the 1.4 Å median RMSD of the original sequences predicted with OmegaFold. (D) Predicted foldability assessed by the predicted local distance difference test (pLDDT) of designed structures (OmegaFold prediction). The dashed line presents the median of 88 pLDDT of the original sequences predicted with OmegaFold.

Next, we assessed the quality of the designed sequences with three *in silico* methods that are independent from both ESM and Rosetta (Figure 3A–D). To test a broad range of metrics, we applied ProteinMPNN^9^ (an inverse folding model using protein backbone coordinates), masked inverse folding with sequence transfer (MIF-ST)^25^ (an inverse folding model using protein atom coordinates combined with a pretrained masked language model) and OmegaFold^26^ (structure prediction requiring no multiple sequence alignment). We have chosen OmegaFold as the authors showed superior performance in single sequence evaluation setting as well as roughly 30-fold runtime improvement compared to other methods. All three design approaches had a similar ProteinMPNN score, with the respective median being very close to the score of the original sequences, with LayerDesign and ESM constraint design having a broader distribution of scores than FixBB (Figure 3A). For the MIF-ST score Rosetta FixBB and LayerDesign had a median pseudoperplexity close to the original sequences, while ESM restrained designs showed a clear improvement (Figure 3B). Lastly, we tested whether the predicted structures of the designs match the target structures and are predicted with high confidence by OmegaFold (Figure 3C,D). All three design approaches generated sequences with low RMSD values to the *de novo* target structures (Figure 3C) and a median pLDDT close to the median of the original sequences. However, for both RMSD and pLDDT the FixBB approach leads to slightly better metrics.

In this work, we combined the predictions of protein language models with structure-based protein design to create sequences with more native-like PLM scores. To do so, we used the ESM language model^11^ to predict amino acid probabilities for Rosetta designed sequences of a benchmark of 34 *de novo* proteins, as these proteins were not part of the language model (PLM) training set. We then restrained the Rosetta energy function with the resulting probabilities and ran an additional round of sequence design. Our main result is that this combination yields sequences with native-like PLM scores, while having little impact on their thermodynamic stability as assessed by the Rosetta energy function and with similar sequence recovery. In contrast, unrestrained Rosetta FixBB design simulations create sequences that have drastically lower PLM scores.

An important aspect of the restrained design approach is the difference between the commonly used PSSM based on evolutionary information and our PSPM based on ESM predictions. While the PSSM represents the independent log-likelihood of observing a particular amino acid at a particular position, our PSPM represents the conditional likelihood given the contextual sequence. Using this distribution without updating the matrix after each mutation does not guarantee generation of sequences that follow the joint distribution of ESM; nevertheless, our ESM restraint designs result in better ESM and MIF-ST scores. We have chosen to not update the PSPM after each mutation as this drastically increases the run time and with this taking away from the benefits of using an PLM.

Analyzing the positions with the worst PLM score in FixBB designs showed a clear bias toward aliphatic surface residues, where a polar charged residue was predicted instead. Amino acid composition biases are a known problem in Rosetta with multiple solutions implemented.^22−24^ One of these solutions is to restrict the available amino acid types at a given position dependent on the solvent-accessible surface area, preventing hydrophobic amino acids at the surface and polar residues in the core of a protein.^22^ Using LayerDesign for our *de novo* benchmark resulted in better PLM scores than the FixBB protocol; however, the resulting sequences still scored worse than their native sequences. Analyzing the worst PLM scoring positions showed a large fraction of polar uncharged residues with PLM proposed replacements to either aliphatic or polar charged residues. As for the FixBB protocol, most of the worst PLM scoring positions were located on the surface. The discrepancy in PLM scores between native sequences and LayerDesign designed sequences highlights that there is not a single rule that perfectly fits all possible protein folds.

To further assess the behavior of the different design approaches, we scored the resulting sequences using ProteinMPNN^9^ and MIF-ST,^25^ as well as predicted their structure using OmegaFold^26^ (Figure 3). For context, the proteins of the *de novo* benchmark were part of the training data of ProteinMPNN, while for MIF-ST and OmegaFold they were not part of the pretraining data of their respective language model but were incorporated during the subsequent stages of training. A direct comparison to sequences sampled by ProteinMPNN is therefore not sensible as potential differences could be influenced by the structure-based approach, but also simply because of the data used during training. Instead, scoring the designed sequences with ProteinMPNN allows for an indirect assessment that describes how well the designs are predicted to fit the particular protein backbones. Interestingly, the FixBB designed sequences did not have significantly worse MIF-ST/ProteinMPNN scores than their original sequences. While restraining Rosetta with ESM predictions did improve the MIF-ST scores, it did not improve ProteinMPNN scores or OmegaFold predictions. We argue that this is due to the ESM being agnostic to the exact target backbone and instead defining a more native-like protein sequence space. In comparison, structure-based methods like Rosetta and ProteinMPNN focus on finding sequences that fold into exactly the desired target structure, often resulting in highly stable proteins existing in a single conformational state. However, evolution balances protein stability and function, with conformational diversity and flexibility being crucial for emergence of the latter. Even in the case of functions requiring very specific geometric constraints, the remaining residues need to support the frustrations imposed by these motifs. This highlights the trade-offs between structure- and sequence-based design which fulfill different protein design and engineering needs and are complementary to each other. Additionally, we have recently shown that restraining Rosetta with evolutionary information leads to a similar impairment of Rosetta energies while retaining residues important for protein function.^27^ In general, Rosetta is perfectly able to design sequences that fold into the given backbone, as illustrated by the fact that some of our *de novo* benchmark cases were the result of the Rosetta protein sequence design in the first place. However, finding the sequence needle in the haystack has historically meant screening many designed protein sequences both computationally and in the wet lab. Comparing and analyzing the differences between Rosetta design protocols and ESM restraint designs highlights the complexity of designing both stable and functional protein surfaces, where hydrophobic patches can lead to misfolding and aggregation. While recently it has been shown that PLMs generalize beyond natural proteins,^28^ it is impressive that PLMs improve even starting from a Rosetta designed sequence with ∼50% sequence identity to the native sequence. For now, designing both stable and functional proteins requires a careful balance of structure- and sequence-based methods, while ideally informing design choices through experimental validation on a case-by-case basis.

A major limitation of our study is the underlying assumption that the PLM score reflects the protein native likeness of a given sequence. As with any model, the resulting scores are an approximation, which might produce scoring sequences that are not native like. However, these models were trained in unsupervised fashion to generalize amino acid probabilities of the UniProt Reference Cluster 50 and multiple recent studies have shown the strong performance of PLMs for a variety of tasks, including protein design where Verkuil et al. showed that the ESM PLM can design sequences for the *de novo* benchmark used in this study by fixing the attention representation.^28^ Additionally, seminal studies by Hie et al. explored how protein PLMs capture evolutionary information which can be leveraged to predict immune-escaping variants of viruses or for the affinity maturation of antibodies.^17,29^ An interesting idea raised by Hie et al. is their manifold hypothesis, that restricting the huge mutational space toward stability (intrinsic fitness) prevents deleterious mutations (“falling of the manifold”) and therefore increases the chances of exploring functional relevant mutations (increasing the extrinsic fitness). This idea is analogous to restricting the mutational space based on evolutionary information, as natural selection has purged highly deleterious mutations, and has been successfully applied to stabilize proteins.^18,19,30^ However, since PLMs only require a single input and generalize beyond their training data,^28^ our approach is also applicable when there is little evolutionary information available yet. Based on this hypothesis, restricting Rosetta design to the predicted likely mutational space of the ESM PLM should aid researchers in engineering and modifying existing proteins by restricting the sequence search space to beneficial mutations. We envision this as an orthogonal approach in addition to structure-based design methods, defining the sequence space to further meet functional needs.

An interesting future work would be to compare the results of ESM restraint designs of natural proteins to the approach of using evolutionary information from a multiple sequence alignment to restrain the Rosetta energy function or to experimentally validate our approach for a particular protein target. In this study, however, we choose to focus on a benchmark of *de novo* proteins as those were not part of the ESM training data and therefore the success was dependent on generalization over the protein space instead of simple memorization of a given sequence or protein family.

## Conclusion

The combination of PLM predictions and structure-based design should aid in the modification of existing proteins as well as in the creation of novel sequences. The potential applications of our work include, but are not limited to, thermostabilizing proteins ranging from enzymes to antibodies and restricting the mutational space toward viable sequences. In conclusion, our work adds a novel way to combine the power of PLM models with the flexibility of Rosetta design protocols through the establishment of the PerResidueEsmProbabilitiesMetric.

## Methods

The scripts used in this work, data sets, and Rosetta runs can be found at github.com/meilerlab/PLM_restraint.

### Collection of a *De Novo* Protein Benchmark

To assess the generalization of protein language models we choose a benchmark of 34 *de novo* proteins, as these proteins were not part of the ESM training data set, based on the recent paper from Verkuil et al.^28^ This data set was chosen as the authors cleaned not only the ESM2 training set of exact sequences but also 58,462 similar sequences. Additionally, it is diverse with respect to both protein length and fold, providing a one-of-a-kind data set. All proteins were relaxed using the FastRelax^31,32^ mover.

### FixBB and LayerDesign Sequence Design Protocols

For each of the 34 benchmark proteins, 1000 sequences were designed using the PackRotamersMover starting from the relaxed structure of each protein. For the LayerDesign protocol,^22^ the available amino acid types were restricted using the LayerDesign task operation in Rosetta and again 1000 sequences were designed using PackRotamersMover.

### Analysis of Protein Language Model Scores of Rosetta Designs

The designed sequences were used as input for the ESM2 650 M model^11,33^ to score each position according to its predicted likelihood and for the ten worst positions the current amino acid and the amino acid mutation with the highest likelihood instead was recorded. For Figure 2A,C the amino acids were grouped as polar charged (Asp, Glu, Lys, Arg), polar uncharged (Gln, Asn, Thr, Ser, Cys), aliphatic (Ala, Ile, Leu, Met, Val), Gly/Pro, His and aromatic (Tyr, Phe, Try). The reported language model score (pseudoperplexity) is the exponential negative average likelihood over the whole protein sequence.

### Incorporation of the Protein Language Model Metric into Rosetta

To facilitate quick scoring and allow protocol development a new PerResidueEsmProbabilitiesMetric was implemented as SimpleMetric^34^ in RosettaScripts using the recently implemented RosettaTensorflowManager.^35^ Therefore, sequence tokenization, model loading, and inference was implemented in C++, including a convenience function outputting predicted probabilities in the format required for the FavorSequenceProfile mover.

### Position-Specific-Probability-Matrix Constraint Rosetta Design

For the ESM constraint designs the best Rosetta total score sequences for each protein of the FixBB protocol were used as input to the ESM2 650 M model,^11,33^ predicting the probabilities of each amino acid at each position. The newly incorporated Rosetta metric returns the probability matrix in the format of a position-specific scoring matrix, which can directly be used with the FavorSequenceProfile mover in Rosetta. The Rosetta energy function was constrained for each protein with the respective probabilities, and again 1000 sequences were designed using PackRotamersMover.

### *In Silico* Validation of Designed Sequences

The pseudoperplexity scores of the resulting designs were scored with MIF-ST^25^ and ProteinMPNN^9^ where the score is the exponential negative average likelihood over the whole protein sequence. We used OmegaFold^26^ (5 recycles) to predict a structure for each designed sequence and the original sequences of the *de novo* benchmark and calculated RMSD.
